# Editorial: Owning a Body + Moving a Body = Me?

**DOI:** 10.3389/fnhum.2019.00088

**Published:** 2019-03-18

**Authors:** Lorenzo Pia, Francesca Garbarini, Andreas Kalckert, Hong Yu Wong

**Affiliations:** ^1^SAMBA (SpAtial, Motor & Bodily Awareness) Research Group, Psychology Department, University of Turin, Turin, Italy; ^2^Neuroscience Institute of Turin, University of Turin, Turin, Italy; ^3^MANIBUS (Movement ANd Body In Behavioral and Physiological Neuroscience) Research Group, Psychology Department, University of Turin, Turin, Italy; ^4^Department of Psychology, University of Reading Malaysia, Iskandar, Malaysia; ^5^Philosophy Department, University of Tübingen, Tübingen, Germany; ^6^Philosophy of Neuroscience (PONS) Research Group, Werner Reichardt Centre for Integrative Neuroscience, University of Tübingen, Tübingen, Germany

**Keywords:** bodily self, sense of agency, body ownership, body representation, self-consciousness

Bodily self-consciousness involves awareness of being the embodied subject of our experience and of residing in and controlling a physical body. Such bodily self-awareness is ubiquitous and stands at the root of human nature (James, 1890). Indeed, sensing the body allows us to distinguish ourselves from the external world and shapes our identity. Currently, there is a wide consensus that the experience of our own body relies on at least two key neurocognitive components (Gallagher, 2000): the sense of agency (i.e., the feeling of authorship over one's own willed actions/thoughts) and the sense of body ownership (i.e., the sense that the physical body is experienced as mine). The sense of agency is thought to be rooted in efference copy mechanisms predicting the sensory consequences of the movements, and the resulting feedback (Haggard, 2008). In contrast, body ownership arises from the integration of body-related afferent signals (e.g., visual, somatic) that constantly reach our own body (Ehrsson, 2012; Kilteni et al., 2015).

Despite widespread agreement that a coherent experience of the bodily self emerges from the complex interplay between the sense of agency and the sense of body ownership, the character, and the form of this bond is not perfectly understood. Indeed, despite the increasing interest in the past years we do not conclusively know to which extent these two experiences interact, at both the behavioral and neural level. It is crucial to understand whether these agency related processes change actually the perceptual processes underlying the sense of ownership.

We have started to examine this relationship more closely, for example by using experimental manipulations of body ownership in healthy participants such as the rubber hand illusion (e.g., Kalckert and Ehrsson, 2012) or through investigating stroke-induced disorders of body awareness (e.g., Pia et al., 2016). This, in turn, has informed philosophical investigations on the bodily self and consciousness (De Vignemont, 2018; Wong, 2018). Our current research topic highlights the need to look at body ownership and sense of agency as a joint process in bodily self-awareness. Here we provide a brief overview of the contributions to the research topic, focusing on general themes that have emerged.

Amongst the non-original research articles, Gallagher defends the phenomenological nature of body ownership from deflationary and eliminativist critiques. He argues that a phenomenological point of view sees ownership to be intrinsic to any experience and that pre-reflective self-awareness subserves ownership. In their meta-analysis of the neuroimaging literature (Zapparoli et al.) show that the three subcomponents of intentionality (i.e., content, timing, and the possibility of generating an action) are anatomically and functionally segregated within the human brain. Lastly, another paper reviews studies of disorders of the bodily self in schizophrenia. Klaver and Dijkerman propose that a weaker internal representation of one's own body can be considered a clinical subcomponent of schizophrenia. This, in turn, would render schizophrenic patients more susceptible to external stimulation.

Our original research articles make use of a variety of approaches, most notably, using virtual reality applications to manipulate perceptual input. Pritchard et al. for instance, implemented the rubber hand illusion paradigm using virtual reality. They investigate how incoming sensory signals affect ownership and agency. The data shows that the visual form, the plausibility, and the spatiotemporal integration of afferent input differentially affects ownership and agency. Similarly, another study (Ratcliffe and Newport) employs a virtual hand illusion paradigm which examines to what extent visual, spatial, and temporal properties of one's own hand affects action embodiment and body ownership. The authors show that visuomotor synchrony is sufficient to trigger agency, but not ownership, for which additional body-related visuospatial information is required. Harjunen et al. employ a virtual bimodal oddball task to study if vision of one's own body affects visuo-tactile interaction in endogenous spatial attention at different levels of visual and somatosensory processing. Results show that seeing one's own body affects cross-modal spatial attention and that this is reflected in early and late-sensory ERPs. Some other original research articles examine different pathological conditions, which affect the experience of one's own body. Rabellino et al. investigate whether traumatic experiences influence bodily perception. The authors administer the rubber hand illusion paradigm in individuals affected by Post Traumatic Stress Disorders (PTSD). The authors found that in PTSD there is a lower susceptibility to the illusion. Alfaro et al. present a rare case of alien hand syndrome in which a patient perceives her arm as having a “mind of its own.” This condition prevented her from playing piano. In the light of the patient's brain damage, which includes the parietal lobe, the authors interpreted this abnormal motor control and anomalous self-body perception as a disruption of efferent outputs. Kanayama et al. examine neuroanatomical variations in relation to a self-report questionnaire measuring general experience of ownership and agency. Their voxel-based morphometry results show a negative correlation between ownership experience and insular gray matter volumes. Kashihara et al. examine the role of body ownership on the emergence of the observation inflation effect (OI)—a false memory phenomenon in which people falsely report having achieved an action when, in fact, they have only observed the action by another person. Indeed, their data shows that bodily self-consciousness has a key role in the emergence of such an effect.

One paper engaged in translational research (Raghavan et al.) attempt to exploit the current knowledge about the bodily self to treat disorders of the bodily self. The authors administered the Music Upper Limb Therapy-Integrated (MULT-I) to stroke patients, who exhibit a variety of symptoms related to the bodily self. They show that this intervention is effective in re-creating patients' sense of self because it allows for the integration of sensorimotor/emotional information and facilitates recovery across multiple domains of disability.

In summary, the present Research Topic suggests that body ownership and sense of agency do not arbitrarily co-occur in our experiences but, rather, can have a significant influence on each other and might even share some neuroanatomical and functional features. Future studies should continue to examine the complex relationship between ownership and agency in healthy as well as pathological conditions affecting bodily awareness. Finally, the development of next-generation prosthetic devices and virtual-reality applications may engender new approaches in neuroscience, rehabilitative medicine, and therapy.

## Author Contributions

All authors listed have made a substantial, direct and intellectual contribution to the work, and approved it for publication.

### Conflict of Interest Statement

The authors declare that the research was conducted in the absence of any commercial or financial relationships that could be construed as a potential conflict of interest.
